# Supplementary material for: Chemical Diversity of Locked Nucleic Acid-Modified Antisense Oligonucleotides Allows Optimization of Pharmaceutical Properties
Source: Mol Ther Nucleic Acids. 2019 Dec 18;19:706–17. doi: 10.1016/j.omtn.2019.12.011 (PMC6965521; doi:10.1016/j.omtn.2019.12.011)
Supplement: Document S1. Figures S1–S7 [file mmc1.pdf]

OMTN, Volume *19*

Supplemental Information

Chemical Diversity of Locked Nucleic Acid-Modified Antisense Oligonucleotides Allows Optimization of Pharmaceutical Properties

Natalia Papargyri, Malene Pontoppidan, Mikael R. Andersen, Troels Koch, and Peter H. Hagedorn

SUPPLEMENTARY FIGURES

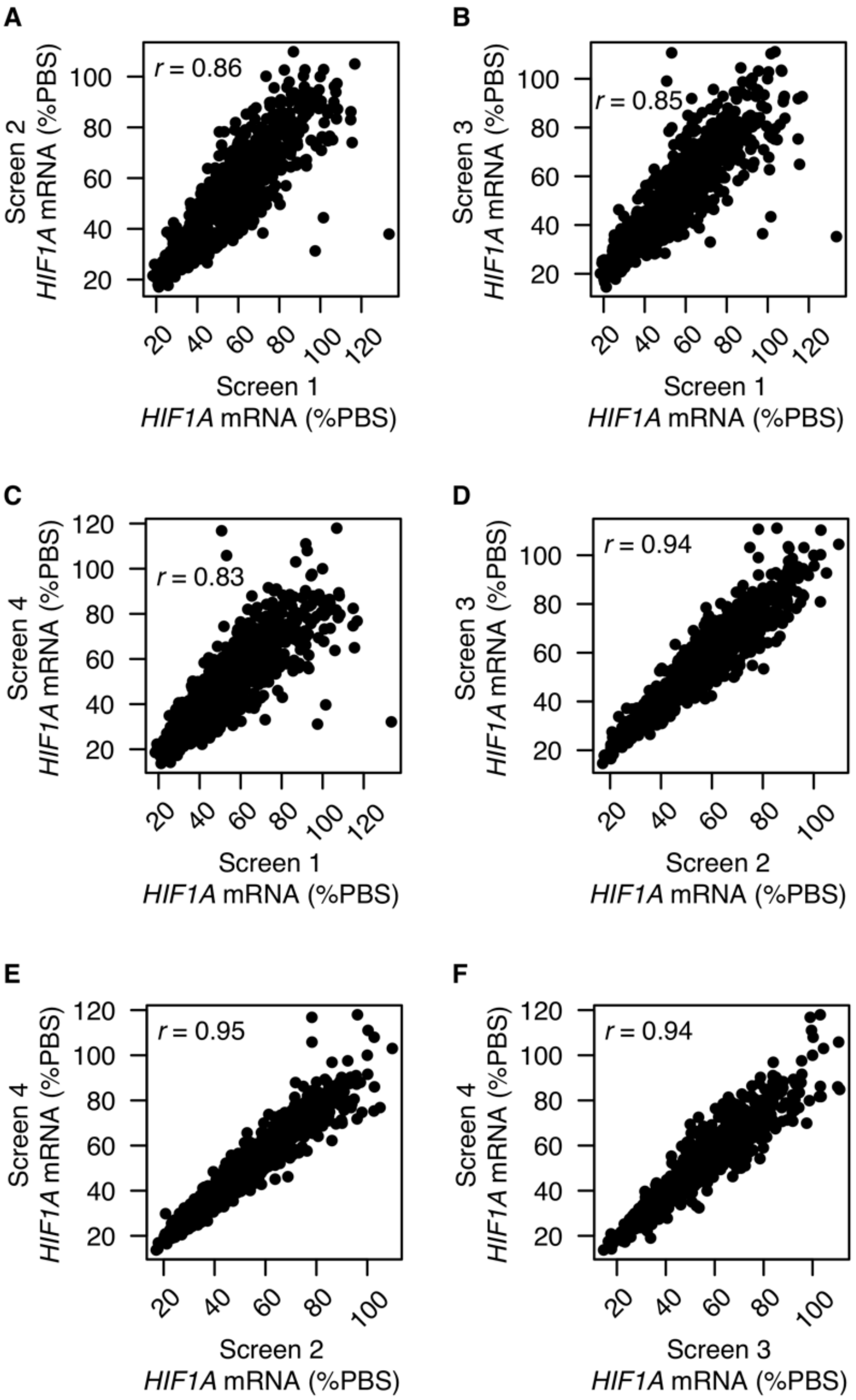

Figure S1. *Reproducible knockdown of HIF1A mRNA across four biological replicate screens*. All 768 LNA-gapmers evaluated in this study are shown. HeLa cells were treated for three days at a concentration of 5 µM by unassisted uptake and mRNA evaluated by RT-qPCR **A)** Screen 1 vs screen 2. **B)** Screen 1 vs screen 3. **C)** Screen 1 vs screen 4. **D)** Screen 2 vs screen 3. **E)** Screen 2 vs screen 4. **F)** Screen 3 vs screen 4. For all scatterplots, *r* indicates Pearson’s correlation.

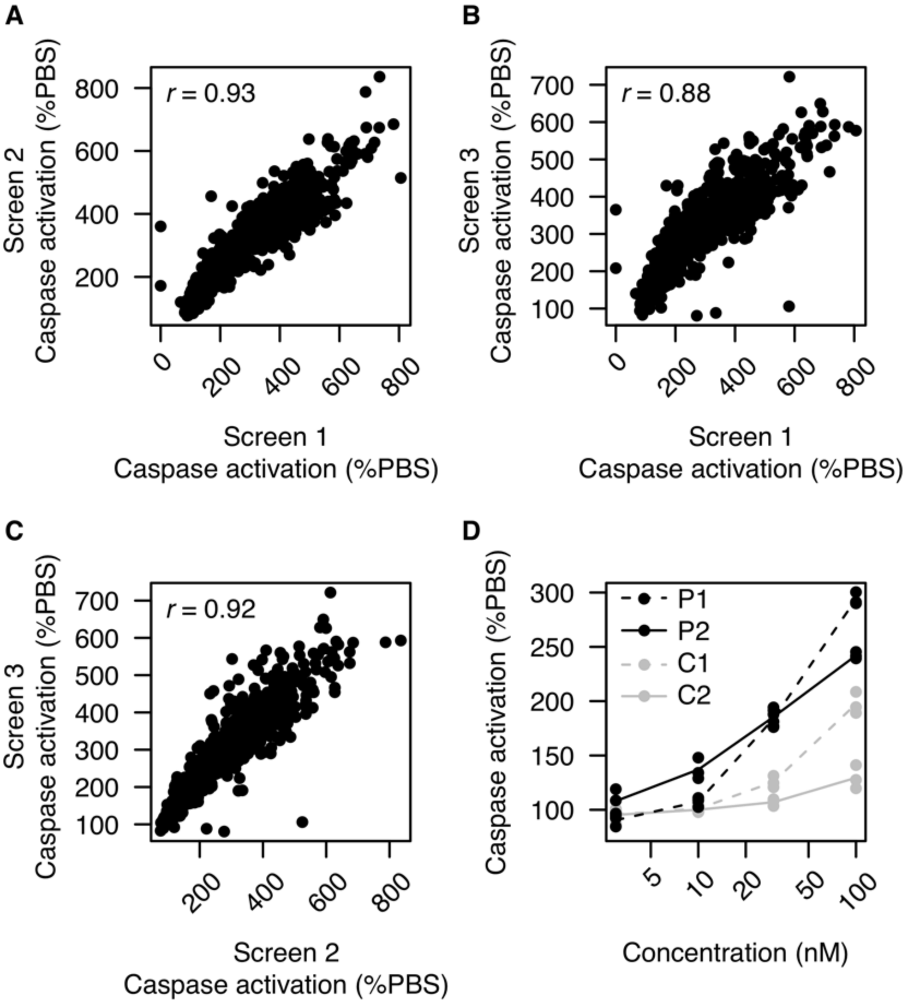

Figure S2. *Reproducible knockdown of caspase activation across three biological replicate screens and evaluation of concentration dependence.* **A)** Screen 1 vs screen 2. **B)** Screen 1 vs screen 3. **C)** Screen 2 vs screen 3. For all scatterplots ABC, *r* indicates Pearson's correlation, and all 768 LNA-gapmers evaluated in this study 24h after transfection of 100 nM in HeLa cells are shown. **D)** Evaluation of caspase activation of two positive control gapmers targeting *HIF1A* and known to knock down *HIF1A* mRNA, P1 and P2, and two negative control gapmers not targeting *HIF1A* and expected to be well-tolerated in cells C1 and C2, see Supplementary Table 1. Four concentrations tested 3, 10, 30 and 100nM. Points indicate individual measurements (*n*=3) and lines indicate average values.

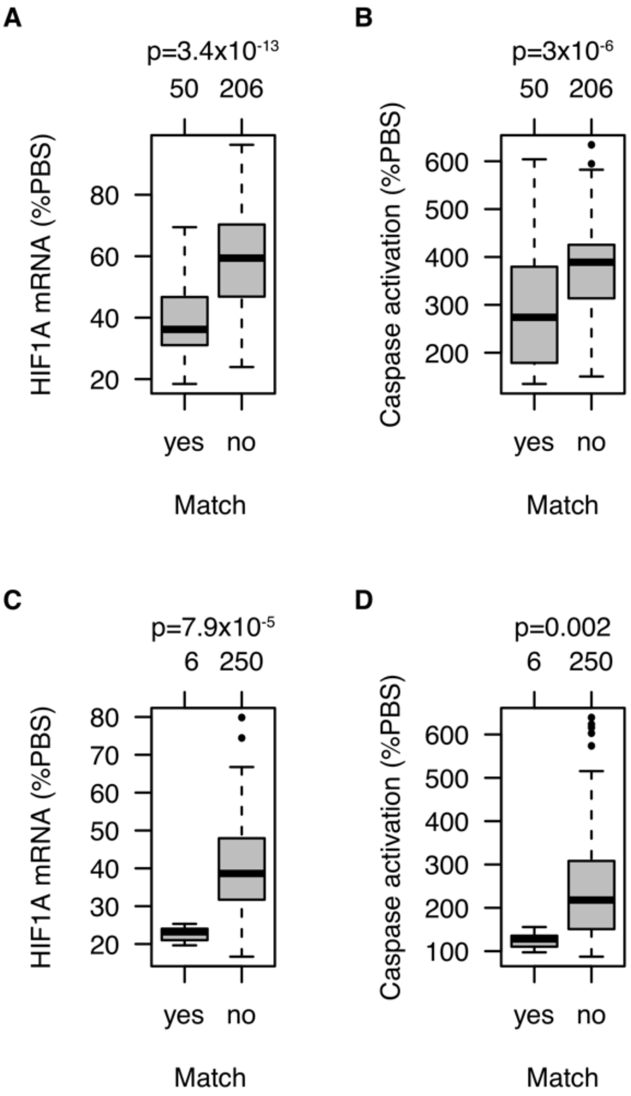

Figure S3. *Preferred chemical modification architectures.* **A)** For region A, boxplots of gapmers matching the preferred criteria (*n*=50 gapmers) with respect to knockdown activity and **B)** cytotoxic potential versus gapmers not matching those criteria (*n*=206). **C)** For region B, boxplots of gapmers matching the preferred criteria (*n*=6 gapmers) with respect to knockdown activity and **D)** cytotoxic potential versus gapmers not matching those criteria (*n*=250). Significance evaluated by two-sided Wilcoxon rank sum test.

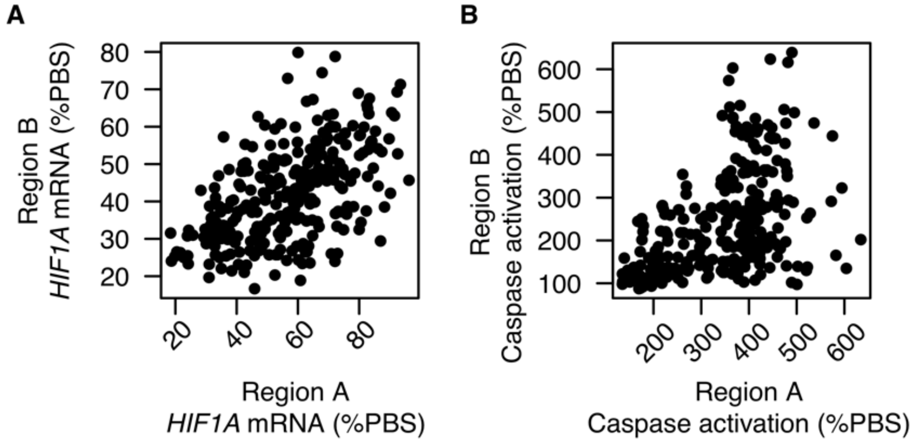

Figure S4. *Comparison of modification architectures between region A and B*. **A)** Knockdown activity. **B)** Cytotoxic potential.

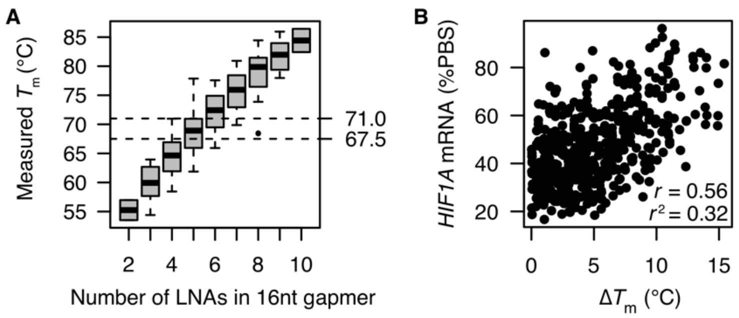

Figure S5. *Impact of LNAs on T_m, and T_m on knockdown activities.* **A)** Boxplots of T_m for 16nt gapmers as a function of the number of LNAs in each gapmer. Dashed lines indicate optimal T_m for regions A and B. **B)** Scatterplot of ΔT_m (absolute difference between gapmer T_m and optimal T_m in target region) versus knockdown activity. *r* indicates Pearson's correlation. r^2 indicates coefficient of determination.

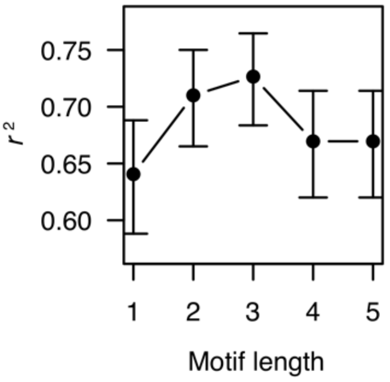

Figure S6. *Proportion of variance explained of knockdown activity of 16nt gapmers by robust linear model as a function of gap motif length in nt.* Error bars indicate 95% confidence intervals.

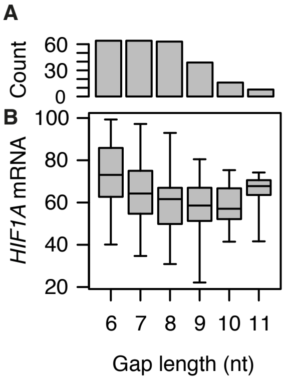

Figure S7. *Measured and predicted knockdown activities for 13nt gapmers stratified by gap sizes*. **A)** Barplot showing number of 13nt gapmers with gap sizes from 6 to 11nt. **B)** Boxplots of measured knockdown activities of *HIF1A* mRNA (%PBS) for 13nt gapmers stratified by gap size.
