# Supplementary material for: Chemical Diversity of Locked Nucleic Acid-Modified Antisense Oligonucleotides Allows Optimization of Pharmaceutical Properties
Source: Mol Ther Nucleic Acids. 2019 Dec 18;19:706–17. doi: 10.1016/j.omtn.2019.12.011 (PMC6965521; doi:10.1016/j.omtn.2019.12.011)
Supplement: Document S2. Article plus Supplemental Information [file mmc3.pdf]

Molecular Therapy
Nucleic Acids
Original Article

Chemical Diversity of Locked Nucleic Acid-Modified Antisense Oligonucleotides Allows Optimization of Pharmaceutical Properties

Natalia Papargyri,[1,2] Malene Pontoppidan,[1,2] Mikael R. Andersen,[1,3] Troels Koch,[2] and Peter H. Hagedorn[2]

[1]Department of Biotechnology and Biomedicine, Technical University of Denmark, 2800 Kgs. Lyngby, Denmark; [2]Roche Pharma Research and Early Development, Therapeutic Modalities, Roche Innovation Center Copenhagen, 2970 Hørsholm, Denmark

The identification of molecules that can modulate RNA or protein function and the subsequent chemical and structural optimization to refine such molecules into drugs is a key activity in drug discovery. Here, we explored the extent to which chemical and structural differences in antisense oligonucleotides, designed as gapmers and capable of recruiting RNase H for target RNA cleavage, can affect their functional properties. To facilitate structure-activity learning, we analyzed two sets of iso-sequential locked nucleic acid (LNA)-modified gapmers, where we systematically varied the number and positions of LNA modifications in the flanks. In total, we evaluated 768 different and architecturally diverse gapmers in HeLa cells for target knockdown activity and cytotoxic potential and found widespread differences in both of these properties. Binding affinity between gapmer and RNA target, as well as the presence of certain short sequence motifs in the gap region, can explain these differences, and we propose statistical and machine-learning models that can be used to predict region-specific, optimal LNA-modification architectures. Once accessible regions in the target of interest have been identified, our results show how to refine and optimize LNA gapmers with improved pharmacological profiles targeting such regions.

INTRODUCTION

In traditional small-molecule drug discovery, large libraries of chemical compounds are screened for activity in cellular assays to identify compound hits that are active against a biological target.[1] High diversity in shape and chemical structure between compounds has been found to increase the probability of finding such hits.[2] The compound hits identified from the initial screening are analyzed for structure-activity relationships with the target and used as structural basis for generation of new libraries. These libraries are then screened for a wider range of properties to identify lead molecules, which may be further optimized structurally before final selection of a clinical drug candidate.[3]

For antisense oligonucleotide drug discovery, we recently proposed a similar process, where diverse libraries are screened to identify oligonucleotide hits, which can then be further chemically optimized until oligonucleotides with acceptable drug-like properties are identified.[4] Antisense oligonucleotides (AONs) are short chemically modified oligonucleotides, designed to bind by Watson-Crick base pairing to a perfectly complementary region in an RNA target of interest and modulate its function.[5,6] We here focus on AONs designed as gapmers with a central gap region of at least six consecutive DNAs to allow the endogenous ribonuclease H1 enzyme (RNase H1) to bind to the heteroduplex between gapmer and target RNA and cleave the RNA strand.[7,8]

The identification of active gapmer hits has historically been based on the so-called gene walk strategy, where many different regions along the RNA are targeted by gapmers with fixed chemical modification architectures, that is, where the types and positions of chemical modifications in the AON are fixed irrespective of the nucleobase sequence. Such fixed architectures include gapmers with five 2′-O-methoxyethyl (MOE) modifications in each flank, and a central gap of 10 unmodified DNAs, the 5-10-5 MOE design,[9] or gapmers employing three or four locked nucleic acid (LNA) modifications in each flank, the 3-10-3 or 4-8-4 LNA designs.[10] It has been a basic assumption in gene walks that the only structural feature of interest between gapmers is their nucleobase sequences.[9] However, results from several recent studies challenge this assumption. For example, when varying both the types and positions of sugar modification in the flank regions of iso-sequential gapmers, marked differences in properties such as half-lives in mouse kidney,[10] hepatotoxic potentials in mouse liver,[11] and allele selectivities in mouse brain,[12] have been observed.

In this study, we set out to further explore the extent to which chemical and structural differences in gapmers can affect their functional properties. We focused our analysis on iso-sequential LNA gapmers and exhaustively varied the number and positions of LNA

Received 8 September 2019; accepted 10 December 2019;
https://doi.org/10.1016/j.omtn.2019.12.011.

[3]Present address: Department of Automation and Digitalization, Novozymes A/S, 2880 Bagsværd, Denmark

Correspondence: Peter H. Hagedorn, Roche Pharma Research and Early Development, Therapeutic Modalities, Roche Innovation Center Copenhagen, 2970 Hørsholm, Denmark.
E-mail: peter.hagedorn@roche.com

 © 2019 The Author(s).
This is an open access article under the CC BY license (http://creativecommons.org/licenses/by/4.0/).

modifications in the flanks, thereby testing 768 gapmers across two different regions in the hypoxia-inducible factor 1-alpha (*HIF1A*) mRNA known to be accessible to gapmers.[13–15] The HIF1A protein is a subunit of the HIF1 transcription factor that regulates cellular and developmental responses to hypoxia and elevated expression of *HIF1A* is associated with poor prognosis in many types of cancer.[13] We evaluated the gapmers in HeLa cells, measuring target mRNA knockdown activity by qRT-PCR and cytotoxic potential by caspase activation. We found that such iso-sequential (but architecturally diverse) LNA-gapmers can have widely different properties and that these differences can be explained partly by how the different architectures impact target binding affinity, as measured by duplex melting temperature, and partly by the presence or absence of particular short motifs in the gap regions.

RESULTS

LNA Modification Architecture and Nucleobase Sequence Affect Knockdown Activity and Cytotoxic Potential in HeLa Cells

We selected two different 16-nt regions on the human *HIF1A* mRNA previously identified as being accessible to gapmers.[13–15] We designed 256 different gapmers targeting each region, varying the number and positions of LNA modifications in each flank. Specifically, we designed all possible combinations of gapmers having either DNA or LNA at positions 2 to 5 from the end of each flank, indicated by x in Figures 1A and 1B, resulting in $2^8 = 256$ different LNA modification architectures targeted to each region.

For each of the 512 gapmers, we treated HeLa cells for 3 days at a concentration of 5 μM by unassisted uptake and evaluated knockdown of *HIF1A* mRNA by qRT-PCR (n = 4). The measured knockdowns were highly reproducible between biological replicates (Figure S1). All data is available as Table S1. As seen in Figures 1C and 1D, for both regions, we observed average knockdown activities ranging from around 20% *HIF1A* mRNA left after treatment, to virtually no effect seen (80%–100% mRNA remaining). Gapmers targeting region A are broadly distributed without any distinct peaks (Figure 1C), whereas for region B, gapmer activities peak at 30%–40% mRNA (Figure 1D). These differences between distributions of knockdown activities in region A and B are highly statistically significant ($p < 10^{-15}$ by Kolmogorov Smirnov test). Similarly, as seen in Figures 1E and 1F, evaluation of cytotoxic potential by measurement

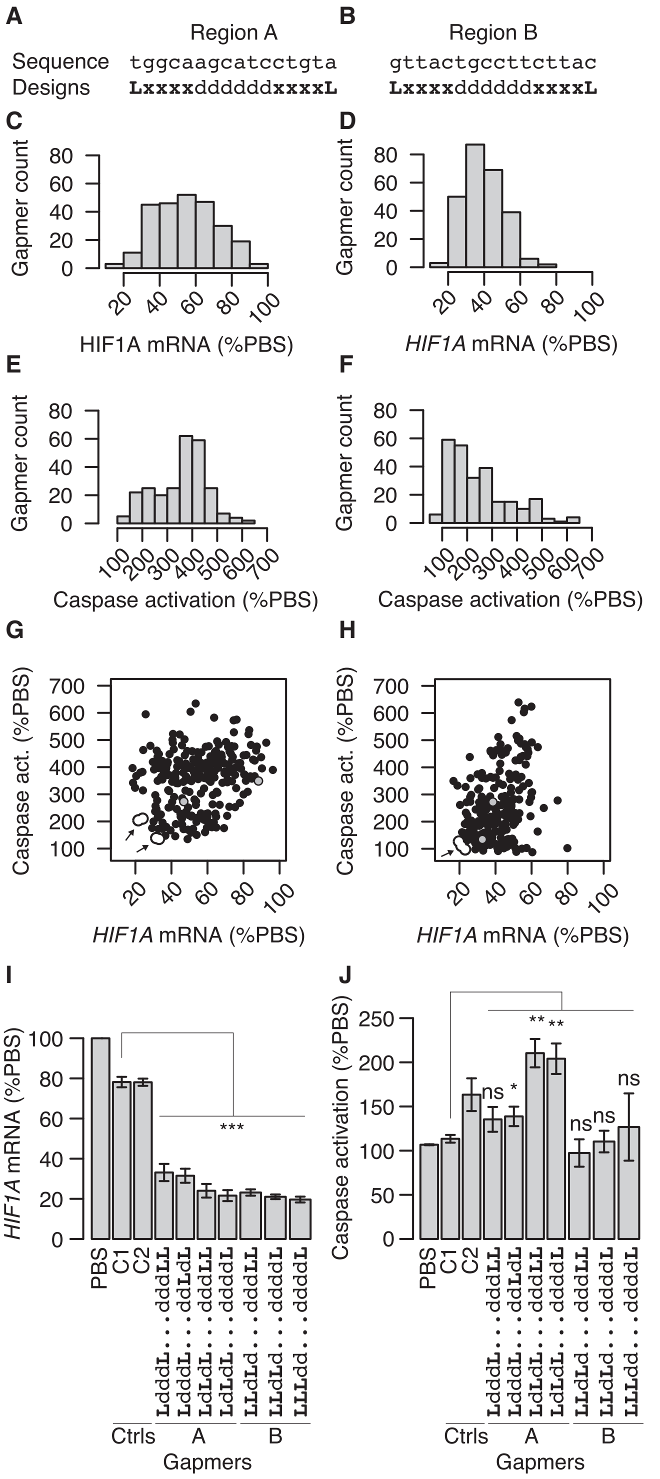

Figure 1. Knockdown Activity and Cytotoxic Potential in HeLa Cells of 16-nt Gapmers Targeted to HIF1A

(A and B) Base sequence and LNA/DNA designs for 256 16-nt-long gapmers targeted to region A (A) and region B (B). L, LNA; d, DNA; x, LNA or DNA. (C and D) Histogram of distribution of knockdown activities for the 256 gapmers targeted to region A (C) and region B (D), quantified as *HIF1A* mRNA levels relative to PBS control after incubation with 5 μM. (E and F) Histogram of distribution of cytotoxic potentials for the 256 gapmers targeted to region A (E) and region B (F), measured by activation of caspase relative to PBS control after transfection of 100 nM. (G and H) Scatterplot of knockdown activity and cytotoxic potential in region A (G) and region B (H). Gray circles indicate the standard 3-10-3 and 4-8-4 designs. Arrows pointing to white circles indicate the top three or four most active and well-tolerated gapmer hits. (I and J) Barplots of knockdown activities of gapmer hits from each region (I), and similarly barplots of cytotoxic potentials (J). Error bars indicate 1 SD. C1 and C2 are negative control gapmers. The variable flank region designs of selected gapmers is shown, with the DNA-only gap region abbreviated to "…." Significance evaluated by Welch t test relative to C1. *$p < 0.05$, **$p < 0.01$, ***$p < 0.001$; ns, not significant.

of caspase activation 24 h after transfection of 100 nM in HeLa cells (n = 3) for both regions resulted in some gapmers activating caspase up to 7-fold compared to PBS controls and others which did not activate caspase. The measured caspase activations were highly reproducible between biological replicates, and concentration dependence was observed when evaluating two negative control gapmers and two *HIF1A* targeting gapmers at four different concentrations, with the largest separation of activities seen at 100 nM (Figure S2). The distributions of caspase activations are highly significantly different between the regions ($p < 10^{-15}$ by Kolmogorov Smirnov test). In region A, there is a peak of gapmers activating caspase at 300%–500%, whereas for region B the peak is at 100%–200%. Since the range of LNA modification architectures are the same in region A and B, the clear differences in distributions of measurements between regions must be a consequence of interactions between these LNA modification architectures and the region-specific base sequences.

For both regions, as seen in Figures 1G and 1H, there was no correlation between knockdown activities and cytotoxic potentials, and consequently, all combinations of activities and cytotoxic potentials were observed. The standard 3-10-3 and 4-8-4 designs are identified by gray circles, and for both regions, many gapmers are more active and better tolerated than these designs. The top three (region A) or four (region B) most active and well-tolerated gapmers are identified by white circles with arrows pointing to them in the lower-left regions of Figures 1G and 1H and are shown in more detail in Figures 1I and 1J. Relative to the two control gapmers, C1 and C2, with three and four mismatches to the *HIF1A* pre-mRNA, respectively (Table S1), a significant 50% to 60% reduction of *HIF1A* mRNA is observed for all seven selected gapmers (Figure 1I). For caspase activation, as shown in Figure 1J, the control gapmer C2 activated caspase 50% relative to C1, which is marginally significant at $p = 0.04$ by Welch's unequal variances t test. Therefore, C1 was chosen as comparison to the seven selected gapmers. Two designs targeting region A and all three designs targeting region B are not or only marginally significantly different from C1 and therefore classed as well-tolerated (Figure 1J). Two designs from region A significantly activate caspase 2-fold relative to C1 (Figure 1J) and are therefore classed as not well tolerated. The LNA modification patterns for the seven gapmer hits are indicated in Figures 1J and 1I as well. For region A, there are two or three LNAs in the 5′ flank, at positions 1, 5, and sometimes 3, counting from the 5′ end. The 3′ flank is shorter, with one or two LNAs at position 1, and sometimes 2 or 3, counting from the 3′ end. There are 50 gapmers that generally fulfill these criteria, and as a group they are significantly more active and better tolerated than the gapmers targeting region A that do not fulfill these criteria (Figures S3A and S3B). For region B, there are three LNAs in the 5′ end, at positions 1, 2, and sometimes 3 and 4. Again, the 3′ flank is shorter, with one or two LNAs at positions 1, and sometimes 2. There are only six gapmers that generally fulfill these criteria, but as a group they are still significantly more active and better tolerated than the gapmers targeting region B that do not fulfill these criteria (Figures S3C and S3D). The LNA patterns of the seven gapmer hits therefore hint at general design criteria that fit well with these two regions, with longer 5′ flanks than 3′ flanks, and only four or five LNAs in total.

Taken together, these results show that varying the number of positions of LNAs in the flanks can dramatically change both knockdown activity and cytotoxic potential (Figures 1C–1F). There are more active and well-tolerated gapmers in region B than in region A (Figures 1G and 1H); however, for both regions, hits can be identified that are highly active and well tolerated (Figures 1I and 1J). Finally, hits can be characterized by having gap regions balanced toward the 3′ end, with four to five LNA modifications in total. For drug development, a typical next step would be to characterize the active and well-tolerated gapmer hits at multiple concentrations to estimate potency and maximal knockdown efficacy from the resulting concentration response curves. This typically allows the overall best gapmers to be identified and progressed. In this study, however, we instead focused on exploring features that can help explain the observed differences in activity and cytotoxic potential between gapmers.

Knockdown Activity Can Be Explained by Presence of Short DNA Motifs in the Gap and Strength of Binding Affinity to the Target Region

None of the LNA modification architectures for the selected gapmers are the exact same between regions A and B, and a systematic evaluation of identical designs between regions reveal no clear correlation with respect to either knockdown or cytotoxic potential (Figure S4). However, the number and positions of LNAs are known to have a substantial effect on binding affinity.[16] To explore how binding affinity influences knockdown activity, we measured melting temperatures (T_m) from absorbance melting curves for all 512 gapmers toward their respective target region. In Figures 2Aand 2B, the knockdown activity as a function of T_m for gapmers in region A and B, respectively, is shown. For both regions, though most clearly for Figure 2B, a parabolic shape is seen, where both too low as well as too high T_m is detrimental to knockdown activity. The T_m range covering the top 10% most active gapmers is indicated with gray bars (Figures 2A and 2B). The midpoint in this range is at 71°C for region A and slightly, albeit significantly, lower, at 67.5°C, for region B ($p < 0.0001$ by Wilcoxon rank-sum test). These estimated midpoints represent optimal T_m values with respect to knockdown activity in each region. We, and others, have also previously reported the existence of such region-specific optimal T_m values for gapmer knockdown activity.[17,18] From this analysis, we also infer that it is not necessarily the actual number of four to five LNAs which characterize active gapmer hits in both regions, as discussed above, but rather the resulting binding affinity between gapmer and target region that those LNAs effect (Figure S5A).

Calculating ΔT_m—defined as the absolute distance between the T_m of each gapmer and the optimal T_m in that region—the Pearson's correlation between ΔT_m and mRNA levels across both regions is 0.56 (Figure S5B). The proportion of variance in the knockdown data explained by T_m alone is therefore at 0.32. That T_m alone only

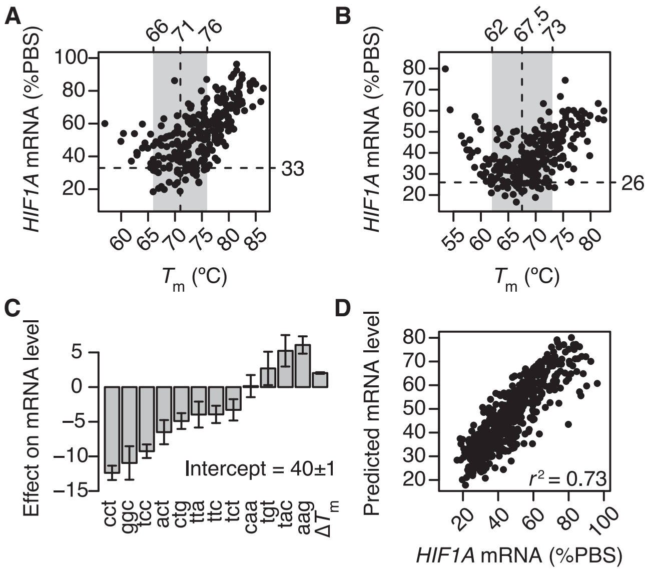

Figure 2. Knockdown Activity Modeled by Duplex Melting Temperature and 3-mer Motifs in the Gap Region

(A and B) Knockdown activity in HeLa cells as a function of temperatures of melting (T_m) measured from absorbance melting curves for all the 256 gapmers targeting region A (A) and the 256 gapmers targeting region B (B). Gray area indicates T_m range for the top 10% most active gapmers. Vertical dashed line indicates midpoint of this T_m range, and horizontal dashed line indicates cutoff for the top 10% most active gapmers. (C) Effect sizes of 3-mer motifs found in the gap regions as well as ΔT_m, the T_m distance from the region-specific optimal T_m, as determined by iterated re-weighted least-squares fitting of a robust linear model. Error bars indicate 1 SD (D). Scatterplot of measured mRNA levels after gapmer knockdown versus mRNA levels captured by the linear model based on 3-mer motifs and ΔT_m. r^2 indicates coefficient of determination.

partly explains the observed knockdown levels can also be seen in Figures 2A and 2B. Even in the T_m-optimal region (gray bars in Figures 2A and 2B), there are many gapmers with low activity. That is, having a T_m close to what is optimal for that target region is a necessary, but not sufficient, requirement for active gapmers.

Considering the mechanism of action of gapmers, after binding to the target RNA, RNase H1 binds to the gapmer/RNA heteroduplex and cleaves the RNA bound within the gap region.[19] The RNase H1 enzyme is known to cleave certain RNA sequences more efficiently than others, because of preference for certain short DNA/RNA heteroduplex sequence motifs over others.[19,20] Taken together with the observation that the gap region is shifted toward the 3′ end of active gapmers, as discussed above, we therefore next explored whether presence or absence of short-sequence motifs can explain the observed variance in knockdown activities. We fitted a robust linear model[21] to ΔT_m as well as to 12 of the possible 19 3-mer DNA motifs found in one or both target regions, modeled as being absent (0) or present (1) (Table S1). The seven motifs not included in this analysis were present in all gapmers from a region and their contributions to knockdown activity therefore not discernable from each other. The fitted effects on mRNA levels of each 3-mer motif as well as ΔT_m is shown in Figure 2C, and the proportion of variance explained is at 0.73, as shown in Figure 2D. Interestingly, only the two 3-mer motifs aag and tac have a clear detrimental effect on knockdown activity (Figure 2C), and they both lie in the 5′ ends of region A and B, respectively (Figures 1A and 1B), and are covered by LNAs in all of the seven gapmer hit architectures (Figures 1I and 1J).

In summary, different LNA modification architectures result in a wide range of knockdown activities (Figures 1C and 1D), and as much three-quarters of this variance can be explained by how close the binding affinity is to the optimal affinity as well as to the presence or absence of certain 3-mer motifs in the gap region (Figures 2A–2D). Both motifs shorter or longer than 3-mers were found to decrease model fits and proportions of variance explained (Figure S6).

Cytotoxic Potential Correlates with Strength of Binding Affinity

The T_m of LNA gapmers have previously been correlated positively with their hepatotoxic potential as observed in mice by measurement of alanine aminotransferase (ALT), as well as to their cytotoxic potential as observed in mouse 3T3 fibroblast cells by measurement of caspase activation.[22] Such a hybridization-dependent cytotoxic potential could be due to increased propensity for off-target effects and generation of RNA cleavage products.[22] For both regions, we also observe that caspase activation generally increases with T_m (Figures 3A and 3B), with Pearson's correlations of 0.55 and 0.78 for regions A and B, respectively. Combining data for the two regions, for all 512 gapmers, T_m correlates clearly with caspase activation and overall explain 0.54 of the variance in the data (Figure 3C).

This correlation between T_m and caspase activation is strong enough to allow a grouping of gapmers into well-tolerated and less well-tolerated gapmers based on a T_m cutoff. About half of all gapmers tested activate caspase more than 300% (dashed line in Figure 3C) and are classed as having a high cytotoxic potential. Similarly, gapmers activating caspase less than 300% are classed as having a low- or medium cytotoxic potential. Using T_m to classify gapmers into those two groups and plotting true positive rates (TPRs) against false positive rates (FPRs) for different T_m cutoffs, the area under the curve (AUC) of the resulting receiver operating characteristic (ROC) curve is at 88% (Figure 3D), demonstrating that T_m allows a both sensitive and specific separation of gapmers.[23] Highest overall accuracy is observed at a T_m cutoff of 73°C (Figure 3E), which separates 80% of gapmers correctly into low/mid- and high-cytotoxic-potential groups (Figures 3E and 3F).

Since for both regions, the optimal T_m with respect to knockdown activity is less than 73°C (Figures 2A and 2B), this allows gapmers to be stratified based on T_m with an acceptable therapeutic window, being both active and well tolerated (compare Figures 2A and 2B with Figures 3E and 3F). Indeed, the four gapmer hits selected from region A (Figures 1G–1J) all have T_m measurements in the range between 65°C and 70°C, and the three gapmer hits selected from region B have T_m measurements in the range between 62°C and 64°C (compare to Figures 2A and 2B).

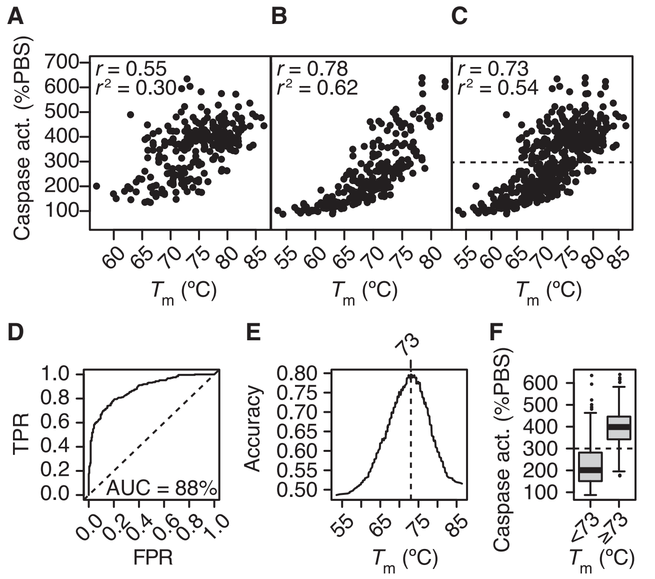

Figure 3. Cytotoxic Potential as a Function of Duplex Melting Temperature
(A–C) Caspase activation in HeLa cells as a function of temperatures of melting (T_m) measured from absorbance melting curves for all the 256 gapmers targeting region A (A), the 256 gapmers targeting region B (B), and all 512 gapmers (C). r indicates Pearson's correlation, r^2 indicate coefficient of determination. Dashed line at 300% indicates suggested cutoff. (D) Receiver operating characteristics curve for a caspase activation cutoff at 300%, varying the T_m threshold. AUC, area under the curve; TPR, true positive rate; FPR, false positive rate. (E) Classification accuracy of gapmers divided into low and high cytotoxic potential (below or above 300%) when varying the T_m threshold. (F) Boxplot showing distributions of caspase activation levels for gapmers divided into a low/mid-T_m group and a high-T_m group based on the optimal T_m cutoff at 73°C.

Properties of 16-nt Gapmers Can Be Learned from Short 13-nt Gapmers Tiled within the Same Region

To independently validate and confirm that measured gapmer properties can be explained by binding affinity and gap motifs, we designed new sets of 128 architecturally diverse LNA gapmers for each region. To allow better stratification of the impact of 3-mer motifs in the gap region, we designed the new sets to be 13 nt in length and tiled them within the 16-nt region covered by the first sets of gapmers (Figures 4A and 4B). In addition, if measured properties of 13-nt gapmers tiled within a region of interest can accurately predict properties of longer gapmers in that region, this could be a resource-efficient manner in which to construct structure-activity relationship models that allow the overall most active gapmers for that region to be identified. To minimize off-target effects, typically, gapmers longer than 13 nt are needed. Most gapmers need to be between 16 and 20 nucleotides in length to achieve acceptable sequence-specificity.[24] This is an additional reason why being able to predict longer, active, gapmers from a set of shorter tool gapmers is desirable.

We evaluated the 13-nt gapmers similarly as was done for the 16-nt gapmers and again observed widespread differences in both knockdown activities (Figure 4C) and cytotoxic potentials (Figure 4D) in both regions. Also, similarly to the 16-nt gapmers, all combinations of activities and cytotoxic potentials were observed (Figure 4E). In contrast to the 16-nt gapmers, the optimal T_m was found to be the same in both regions, at 66°C (compare Figures 4F and 4G with Figures 2A and 2B), albeit this estimate becomes more uncertain as fewer gapmers are included. The T_m that most accurately groups 13-nt gapmers into high- and low/mid-cytotoxic potentials was identified in the same way as for 16-nt gapmers to be at 68°C (Figures 4H–4J). Also, in this case, it allows an 80% accurate classification (Figures 4J). This T_m threshold at 68°C is close to the optimal T_m for knockdown activity at 66°C, making it more difficult to identify active and well-tolerated 13-nt gapmers, and it is 5°C lower than for the 16-nt gapmers (compare Figures 3E and 4J).

Overall, the 13-nt gapmers display the same range of properties and relations between observables as the 16-nt gapmers. We therefore trained statistical models for both knockdown activity and cytotoxic potential, using only the data generated for the 13-nt gapmers, in order to test whether such trained models could predict the properties observed for the 16-nt gapmers. We first trained a robust linear model on the mRNA levels observed after treatment with the 13-nt gapmers similarly as was done above. When using the trained activity model to predict mRNA levels after treatment with the 16-nt gapmers, we observed strong correlation between predicted and measured observables (Figure 4K). At a cutoff of < 50% mRNA remaining, the trained activity model achieved an AUC of 91%. Second, inspired by previous work on predicting hepatotoxic potentials of gapmers,[25] we trained a regression random forest model[26] on caspase activation levels, with T_m and counts of dinucleotides across the entire gapmer as variables in the model. When using the trained toxicity model to predict caspase activation levels after treatment with the 16-nt gapmers, also in this case did we observe strong correlation between predicted and measured observables (Figure 4L). At a cutoff of < 300% caspase activation, the trained toxicity model achieved an AUC of 92%. Previous work suggests this as a reasonable cutoff to separate well-tolerated from hepatotoxic gapmers.[22] The white dots in Figures 4K and 4L indicate the seven gapmer hits identified in Figures 1G–1J. These seven hits are part of a larger group of 107 16-nt gapmers predicted to be both active (< 50% mRNA remaining) and reasonably well tolerated (< 300% caspase activation; gray dots in Figures 4K and 4L). That is, out of the 512 possible 16-nt LNA-modification architectures, the trained models allow identification of a subset of 20% of them, which still includes all the gapmer hits.

When inspecting the fitted effects on mRNA levels of each 3-mer motif, as well as ΔT_m for the 13-nt gapmer activity model (Figure 4M), most of the parameters roughly correlate with the effect sizes identified from fitting the 16-nt gapmers (Figure 4N and compare Figures 4M and 2C). The only notable difference is found for the 3-mer motif ggc, with an estimated effect size at −11 when fitting 16-nt gapmers (Figure 2C) but at +3 when fitting 13-nt gapmers (Figure 4M).

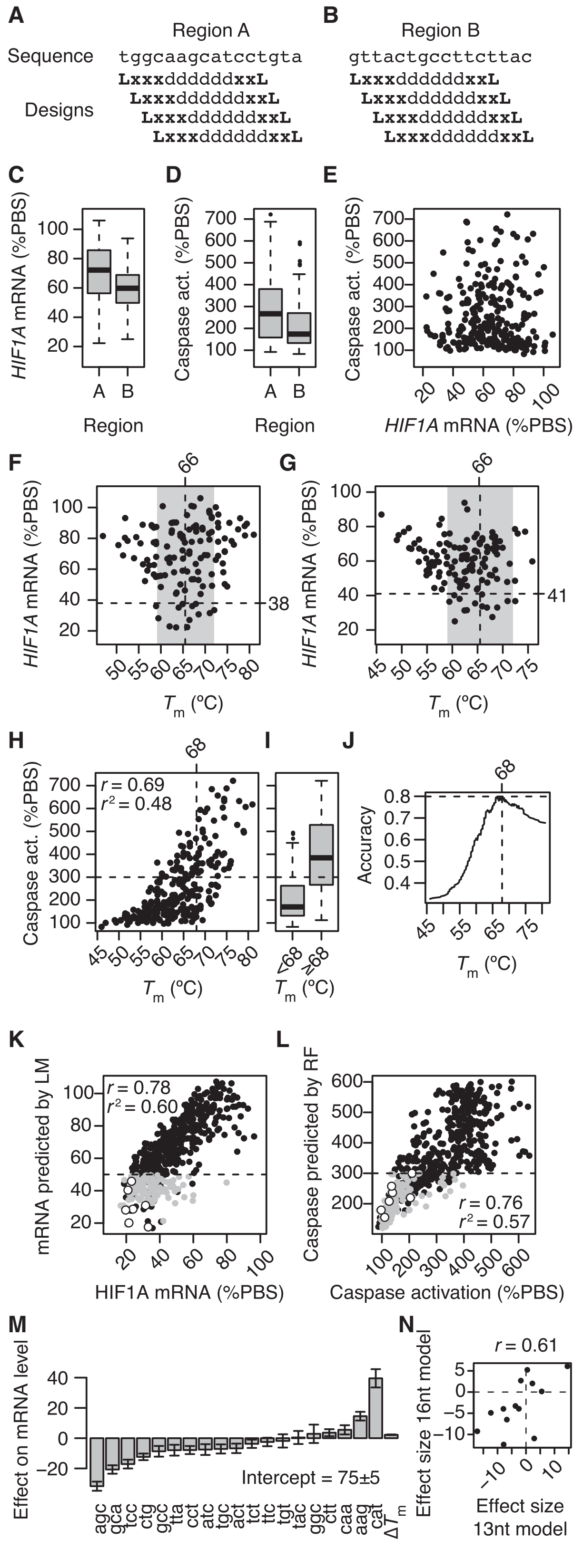

Figure 4. Evaluation of Tiled 13-nt Gapmers and Prediction of 16-nt Gapmer Properties

(A and B) Base sequence and LNA/DNA designs for 4 × 32 = 128 gapmers, 13 nt in length, tiled within region A (A) and region B (B). L, LNA; d, DNA; x, LNA or DNA. (C) Histograms of distributions of knockdown activities for the 2× 128 13-nt gapmers targeted to region A and B, respectively. Quantified as *HIF1A* mRNA levels relative to PBS control after incubation with 5 μM. (D) Histograms of distributions of cytotoxic potentials for the 256 gapmers targeted to region A and B, respectively. Quantified by activation of caspase relative to PBS control after transfection of 100 nM. (E) Scatterplot of knockdown activity and cytotoxic potential in both regions A and B. (F and G) Knockdown activity in HeLa cells as a function of T_m for the 128 13-nt gapmers targeting region A (F) and the 128 13-nt gapmers targeting region B (G). Gray area indicates T_m range for the top 10% most active gapmers. Vertical dashed line indicates midpoint of this T_m range, and horizontal dashed line indicates cutoff for the top 10% most active gapmers. (H) Caspase activation in HeLa cells as a function of T_m for all 256 13-nt gapmers targeting region A and B. *r* indicates Pearson's correlation, r^2 indicate coefficient of determination. Horizontal dashed line at 300% indicates suggested cutoff. Vertical dashed line indicates T_m cutoff achieving best classification accuracy. (I) Boxplot showing distributions of caspase activation levels for gapmers divided into a low/mid-T_m group and a high-T_m group based on the optimal T_m cutoff at 68°C. (J) Classification accuracy of gapmers divided into low and high cytotoxic potential (below or above 300%) when varying the T_m threshold. (K) Scatterplot of measured versus predicted mRNA levels for 16-nt gapmers. Predictions made by robust linear model based on 3-mer motifs and ΔT_m trained from 13-nt gapmers. (L) Scatterplot of measured versus predicted caspase activation levels for 16-nt gapmers. White dots indicate the most active and well-tolerated gapmers identified, and gray dots indicate 16-nt gapmers predicted to be both active and reasonably well-tolerated. Predictions made by random forest model based on dinucleotide motifs and ΔT_m trained from 13-nt gapmers. (M) Fitted effects on mRNA levels of 3-mer motifs in the gap regions, as well as ΔT_m for the 13-nt gapmer activity model, the T_m distance from the region-specific optimal T_m, as determined by iterated re-weighted least-squares fitting of a robust linear model. Error bars indicate 1 SD. (N) Correlation between effect sizes identified from fitting the 13-nt gapmers with those from the fitting of 16-nt gapmers.

That is, irrespective of length, iso-sequential but architecturally diverse LNA gapmers have widely different properties, and these properties can be explained partly by how the different architectures impact target binding affinity and partly by the presence or absence of particular motifs in the gap regions. With enough gapmers, as shown here, these relations can be learned and used for accurate predictions with very high-class separation capacity (AUCs > 90%).

The T_m of LNA gapmers can be predicted using a nearest neighbor model for LNA-modified oligonucleotides approximated from publicly available thermodynamic parameters.[16,17] Training the 13-nt activity and toxicity models on predicted T_m instead of experimentally measured T_m, the class separation capacity when predicting 16-nt gapmers drops to an AUC of 79% for the activity model and to 91% for the toxicity model. That is, in particular for the activity model, an accurate assessment of binding affinity is needed for accurate predictions of activity.

Identification of Optimal Gap Size for Knockdown Activity

It has previously been reported that LNA-gapmers with gap regions of only 4 to 5 nt resulted in no appreciable cleavage of target RNA

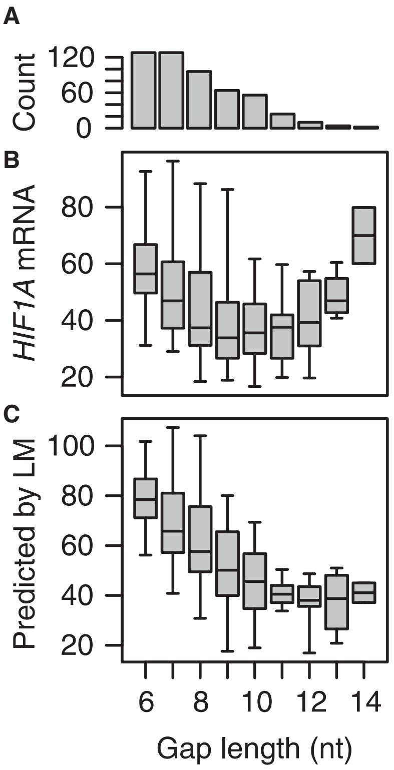

Figure 5. Measured and Predicted Knockdown Activities for 16-nt Gapmers Stratified by Gap Sizes

(A) Barplot showing number of 16-nt gapmers with gap sizes from 6 to 14 nt. (B) Boxplots of measured knockdown activities of *HIF1A* mRNA (%PBS) for 16-nt gapmers stratified by gap size. (C) Boxplots of predicted knockdown activities for 16-nt gapmers stratified by gap size.

when evaluated by *E. coli* RNase H cleavage assay, whereas gaps from 6 to 8 nt resulted in progressively more extensive cleavage of the RNA.[27] It has also been shown that the longer the gap, the faster the degradation of LNA gapmers by endonucleases.[28] These opposing mechanisms suggest the existence of an optimal gap size for LNA gapmers. The 13- and 16-nt gapmers evaluated in this study had gap sizes ranging from 6 to 11 nt (Figure S7A) and 6 to 14 nt, respectively (Figure 5A). For the 16-nt gapmers, we observed that gapmers with lengths between 9 and 11 nt generally resulted in the most activity (Figure 5B), which could be explained by opposing mechanisms such as those suggested above. Similarly, for the 13-nt gapmers, the optimum was found to be between 9 and 10 nt (Figure S7B).

When stratifying the activity model predictions for the 16-nt gapmers by gap size, we also observed that shorter gaps in general resulted in less active gapmers than longer gaps (Figure 5C). Assuming that the 3-mer motif effects trained in the model captures the preference of RNase H for some sequences over others,[20] the modeling predictions consequently support the explanation that, in general, the fewer regions there are to potentially support RNase H binding and cleavage, the lower the activity. However, in contrast to the experimental data (Figure 5B), for the model predictions, the activity reaches a floor at around 11nt and remains at this level also for longer gap sizes (Figure 5C). This could be explained by the model not capturing a gap-size-dependent endonuclease susceptibility and suggests directions for future modeling work.

DISCUSSION

The medicinal chemist tasked with developing molecules with drug-like properties faces the three challenges of distribution, activity, and tolerance. That is, being able to engineer molecules that can get from the point of delivery to the intended tissue and cellular site of action are sufficiently active on the intended target to have pharmacological effect and have no or acceptable off-target effects.

For AON drug discovery, sufficient knockdown activity is reached when the gapmer can bind to the target RNA to an extent that allows the endogenous RNase H1 enzyme to cleave enough of the RNA to have pharmacological effects. To achieve this, first, regions on the target RNA that can be engaged with gapmers must be identified. For example, it is well known that RNA can fold into structures such as stems and various kinds of loops, and although recent studies suggest that mRNAs tend to be relatively unfolded inside cells,[29] regions in RNA with a propensity to be structured will be less accessible to gapmers compared to unstructured regions.[30] Proteins bound to the RNA can also be expected to affect accessibility in those regions.[31] In addition, the nucleobase sequence in accessible regions are required to be unique to the RNA target and not found in any other RNAs, so that gapmers specifically bind and facilitate cleavage of the intended target.[24] Second, gapmers must be identified that target an accessible region and are both active and well tolerated. For example, even when a gapmer targets an accessible region, the available gap region might preclude effective binding of RNase H,[20] the binding affinity to the RNA may be too strong or too weak for efficient turnover,[17,18] or it may cleave unintended RNA or support binding of proteins that elicit cytotoxic effects.[32]

Historically, AON drug discovery has been focused on identifying accessible regions on target RNA using gapmers with one or only a few standard modification architectures.[9,10] If none of the few gapmers tested in a region were sufficiently active and well tolerated, that region was abandoned. A few studies have more recently suggested that a wide range of properties can be achieved when changing modification architectures of iso-sequential gapmers engaging the same region on an RNA target of interest.[11,12] These studies have hinted at the possibilities of leveraging the diversity in chemical modification architectures to optimize key drug properties but have not been comprehensive or systematic enough to allow structure-effect modeling by application of machine-learning algorithms.

In this work, we selected two regions in the human *HIF1A* mRNA already known to be accessible to gapmers and focused on the challenges of engineering gapmers that are both active and well tolerated. By measuring both of these properties in parallel for all molecules, we expected to be able to identify those that have an

appropriately balanced activity-tolerance profile.[33] Presumably, any change in the chemistry and structure of the gapmer could also affect its distribution, although often gapmers can be covalently conjugated with, for example, carbohydrate- or fatty-acid ligands that facilitate effective uptake into certain tissues such as liver or muscle, irrespective of base sequence and chemical modification architecture.[34,35]

We focused on systematically evaluating all possible LNA modification architectures for gapmers 16 nt in length, while leaving at least six consecutive DNA nucleotides in the gap region to support RNase H binding.[19] For both of the selected regions, we identified many gapmers that were both more active as well as better tolerated than the standard LNA modification architectures often used[10] and many that were not.

We evaluated gapmer effects in HeLa cells. Knockdown was measured by qRT-PCR 3 days after unassisted uptake of gapmer into cells without transfection reagent, whereas cytotoxic caspase activation was measured by enzymatic assay 24 h after transfection of gapmer into cells. These assays and protocols have each been suggested previously for evaluating these properties,[22,32,36] but depending on the intended target tissue and delivery method, other activity and tolerance assays or animal models may be more relevant and translate better to humans. For gapmers, the level of caspase activation in HeLa cells after transfection have been correlated with their hepatotoxic potential in mice as evaluated by increased serum levels of ALT observed 3 days[32] or 2 weeks[22] after first dosing. These studies together only evaluate 14 different gapmers, and more studies are needed to establish the extent of the general predictivity of this *in vitro* assay for the hepatotoxicity observed in mice. In this study, we merely assert that chemical modification architectures will significantly impact measured properties irrespective of the assays used to evaluate activity and tolerance. We expect, however, that the relation between these properties and gap motifs and duplex binding affinity is likely to differ between cell types and even between different regions on the same target. We therefore consider the machine-learning models trained in this study to be local, in the sense that their applicability domains may only be for those two regions on the *HIF1A* mRNA in HeLa cells. Interestingly, for both regions, the gapmer hits identified (Figures 1I and 1J) have longer 5′ flanks than 3′ flanks. One reason for this could be that the preferred RNase H binding and cleavage regions are at the 3′ end of the gapmer, or it could be that for optimal multiple-turnover capability, after cleavage the two RNA fragments should have similar binding affinity to their respective parts of the gapmer, so that they drop off with similar rates.[17,18]

It has previously been reported that the presence or absence of certain short sequence motifs in gapmers can be correlated with their knockdown activity.[37] This, as well as many other gapmer- or target-derived parameters, have been used as input variables to machine-learning models for knockdown activity.[38,39] Previous model work, however, has been based on smaller numbers of gapmers of various modification chemistries collected across many different studies with widely different experimental setups. Here, we systematically varied only LNA modification architectures for gapmers tiled within target regions known to be accessible on the same target and evaluated properties under the same experimental conditions. We chose to model knockdown activity as a simple linear combination of ΔT_m and 3-mer gap motifs to better understand how individual motifs affected mRNA levels. We controlled for outliers and unequal variances using robust regression by using a generalization of maximum-likelihood estimation, iterated re-weighted least-squares, when fitting the linear model.[21] Other learning models may be able to achieve higher predictive accuracy. However, irrespective of the data model chosen here, it may be possible to generalize our methodology. Future work could focus on generating sufficient data from different targets and target regions, using standardized experimental protocols such as those proposed here, to allow globally predictive models to be developed. To achieve this, we speculate that other key parameters besides T_m and gap motifs may be necessary to measure as well, such as susceptibility to endonuclease degradation or target-specific transcription rates.[24] Also, as outlined in this study, libraries of gapmers designed as training sets for such models should systematically vary the modification architectures that the models are expected to learn.

In this work, we measured T_m for all gapmers and used those measures as part of the input to the prediction models of activity and toxicity. The T_m is a standard and widely used experimental measure of duplex stability, although not strictly proportional to binding affinity at physiological conditions.[40] Instead, the change in standard free energy of binding, $\Delta G°$ at 37°C, is a more physiologically relevant representation of binding affinity, since $\Delta G°$ is logarithmically proportional to the equilibrium constant between free and duplexed oligonucleotides.[41] Practically, to calculate $\Delta G°$ by van ‘t Hoff analysis, evaluation of T_m at, typically, more than 10 different concentrations are needed.[40] This was judged as unfeasible in the present study covering 768 gapmers. Still, we might have observed more accurate model predictions had $\Delta G°$ been available as input to the models instead of just T_m.

In order to develop prediction models that do not rely on such experimentally measured input, future work could focus on establishing nearest-neighbor models for binding affinity of LNA gapmers with full phosphorothioate backbones binding to RNA that are accurate enough to substitute for measured values. Our results show that this is needed in particular for the activity model, where we observed a > 10% drop in AUC for the ROC curve when using an approximate nearest-neighbor prediction of LNA gapmer T_m[17] instead of measured values. Establishing an accurate nearest-neighbor model for binding affinity prediction would have the additional benefit of also being a straightforward way to replace T_m by $\Delta G°$, as discussed above, since both can be calculated with equal ease with such models.[17]

Several studies have observed that combining different sugar-modification chemistries can reduce cytotoxic potentials, as measured by

caspase activation in cells and hepatotoxic potential in mice.[11,32] For example, combining LNA and 2′-O-methyl (2′-OMe) sugar modifications can lead to reduced cellular protein binding to gapmers with full phosphorothioate backbones and mitigate toxicity in HeLa cells and in mice.[32] Future work could focus on exploring such interaction effects in a systematic fashion as outlined here. However, the additional number of gapmer combinations is substantial. For example, for 13-nt gapmers with five variable sites in the flank regions at which DNA, LNA, or 2′-OMe could be placed, there are $3^5 = 243$ combinations. Four 13-nt gapmers tiled in a 16-nt region gives $4 \times 243 = 972$ gapmers, compared to the 128 gapmers needed when only DNA or LNA is considered. Taking the broad range of cytotoxic potentials seen in this study as a benchmark for the breadth of what can be achieved by varying just the LNA modification architecture, it will be interesting to explore whether combinations of sugar chemistries extend this range or merely provide a different way to realize it.

In our data, we also see that shorter gap sizes result in less active gapmers compared to longer gap sizes. Accordingly, the more 3-mers available for RNase H to bind, the higher the expected activity in general. This relation is captured by our activity-model predictions, demonstrating that only two factors are needed to explain this behavior: T_m and 3-mer contributions. When gap sizes become too long, we also observe less active gapmers. However, this relation is not captured by our model predictions, suggesting other factors not included in the model are involved. One possibility could be increased susceptibility to endonuclease degradation as a function of gap width.[28]

In conclusion, we have systematically demonstrated how small changes in chemical modification architecture can have large effects both on knockdown activity and cytotoxic potential, leading to gapmers with improved pharmacological profiles. This suggests that once accessible and unique regions on the target RNA have been identified, libraries of gapmers that span a diverse range of chemical modification architectures may be essential for discovering gapmers with truly drug-like properties. Therefore, considerations, such as which architectures to evaluate and when, will be important when planning the drug discovery screening strategy. Our results show that by comprehensively evaluating all possible modification architectures within a certain region of chemical space (here LNA modifications), accurate structure-effect models can be trained, allowing systematic optimization of drug-like properties.

MATERIALS AND METHODS

Oligonucleotide Synthesis and Purification

All 768 *HIF1A* LNA-modified gapmers and controls with complete phosphorothioate backbones were synthesized on a MerMade 192× synthesizer (Bioautomation, Texas, USA) following standard phosphoramidite protocols. The final 5′-dimethoxytrityl (DMT) group was left on the oligonucleotide. Following synthesis, the oligonucleotides were cleaved from the controlled-pore glass (CPG) solid support by means of aqueous ammonia and subsequently deprotected at 65°C for 5 h. The oligonucleotides were purified by solid phase extraction in TOP cartridges (Agilent Technologies, Glostrup, Denmark) using the DMT group as lipophilic handle and chromatographic retention probe for purification purposes. The DMT group was removed after the elution of impurities. As the last purification step, the oligonucleotides were eluted from the cartridge and evaporated to dryness. The oligonucleotides were dissolved in Dulbecco's PBS (DPBS) (D8537; Sigma, Søborg, Denmark), and the concentration of oligonucleotide in solution was determined by calculating the Beer-Lambert extinction coefficient and measuring UV absorbance. Oligonucleotide identity and purity were determined by reversed-phase ultra performance liquid chromatography coupled to mass spectrometry (UPLC-MS).

Cell Lines

Human epitheloid cervix carcinoma HeLa cells (93021013, ECACC through Sigma) were cultured in Eagle's minimum essential medium (EMEM) with Earle's salts (M2279; Sigma) supplemented with 10% fetal bovine serum (FBS) (F7524; Sigma), 2 mM glutamine, 0.1 mM (1×) non-essential amino acids (NEAA) (M7145; Sigma), and 25 μg/mL gentamicin (G1397; Sigma). The cells were grown at 37°C with 5% CO_2 in a Cytomat 10°C humidified automated incubator (Thermo Fisher Scientific). In all experiments, the cells were subcultured a minimum of five and maximum of 15 times, counted from the day of thawing.

mRNA Reduction after Unassisted Uptake by qRT-PCR

HeLa cells were plated in 96-well plates at a cell density of 2,500 cells/well in 95 μL of growth media. The cells were incubated for 24 h before the addition of gapmers, achieving a total volume of 100 μL and a final concentration of 5 μM. Cell plates were harvested 3 days after their exposure to gapmers, and RNA was purified using QIAGEN RNeasy 96 kit (74192; QIAGEN) according to manufacturer's guidelines. Freshly eluted RNA plates were kept on ice to avoid unwanted reverse transcriptase (RT) enzyme activity. RNA yield was measured on an EON microplate reader (BioTek, Winooski, VT, USA). The RNA was diluted into new RNA dilution plates (AB0900; Thermo Fisher Scientific), so that the final RNA concentration was within the range of 0.2–5 ng/μL. The plates were sealed, heat-shocked for 40 s at 90°C to melt RNA:gapmer duplexes, and were then put on ice directly. A one-step mRNA quantification by quantitative reverse transcription PCR (qRT-PCR) protocol (cDNA synthesis and amplification steps combined in one) was followed in this study. The *HIF1A* Taqman probe (Hs00936368_m1; Thermo Fisher Scientific) was labeled with FAM-MGB dye, and the Taqman probe for the reference gene, beta glucurodinase (GUSB) (4326320E; Thermo Fisher Scientific), was labeled with VIC-MGB dye. A second reference gene was also used for the first screen; however, as its performance was very similar to that of GUSB and their average was equal to that of GUSB alone, therefore, we continued only with one reference gene. These probes were run in a duplex system, with qScript XLT one-step RT-qPCR ToughMix, low ROX (95134-500; Quanta Bioscience) as assay buffer. A master mix of the above was made by mixing 0.5 μL of each probe in 5 μL of buffer per reaction,

and reaction was loaded twice (duplicates). The volume of 6 μL of master mix per well was added on MicroAmp optical 384-well plates (4309849; Applied Biosystems). 4 μL of diluted RNA was added per well to achieve 10 μL total volume per reaction. The plates were sealed with MicroAmp optical adhesive film (4311971; Applied Biosystems), vortexed so that XLT buffer is again adequately mixed with the diluted RNA, and finally spun down for 3 min at maximum speed. The plates were run in a ViiA 7 real-time PCR system with an OptiFlex optics system (Thermo Fisher Scientific), using the following program: 15 min at 50°C, 3.5 min at 95°C, followed by 40 cycles of 95°C for 5 s and 60°C for 45 s. The set temperature change rate is 1.9°C per second for all steps. Standard curves were included as performance controls in all runs.

Measurement of Caspase Activation after Transfection

HeLa cells were plated in growth medium without antibiotics (100 μL/well) 1 day prior to transfection in white 96-well plates with clear bottom (6005181; Perkin-Elmer). Inoculation cell density was 4,000 cells/well such that they would be 30%–50% confluent at the time of transfection. On the day of transfection, the HeLa cell plates were assessed for cell viability under a microscope (CKX41SF; Olympus Life Science Denmark). Then, each LNA-modified AON was diluted in 25 μL Opti-MEM I (1×) + GlutaMAX-I reduced serum medium (51985-026; Gibco) by mixing gently to achieve final concentrations of 100, 30, 10, and 3 nM; each concentration in triplicates per sample. Lipofectamine 2000 reagent (11668-019; Invitrogen) was mixed gently by inverting before use and a Lipofectamine/Opti-MEM master mix was subsequently prepared by gently mixing 0.25 μL Lipofectamine 2000 in 25 μL Opti-MEM per sample and left to incubate for 5 min at room temperature. After a 5-min incubation, diluted LNA-modified AONs were combined with diluted Lipofectamine 2000 (25 μL Lipofectamine 2000/Opti-MEM complex mixed gently in 25 μL of diluted LNA-modified AONs) and left to incubate for 20 min at room temperature. In the meantime, growth medium was aspirated from the HeLa cell plates using low vacuum, and 50 μL of Opti-MEM was added per well. After the 20-min incubation, 50 μL of each LNA-modified AON/Lipofectamine 2000 complex were added onto the HeLa cells and were mixed by rocking the plate gently back and forth to ensure distribution of the LNA-modified AON/Lipofectamine 2000 complexes. Plates were transfected with 5-min intervals between each, in order to ensure equal time between the transfection point and caspase quantification (see below). The HeLa cell plates were left to incubate at 37°C in a CO_2 incubator for 4 h. After the 4-h incubation, 50 μL of growth medium supplemented with 30% FBS (medium with antibiotics) were added to each well, and plates were mixed gently by rocking back and forth (total volume per well, 150 μL). The day after transfection, in good time to prepare for caspase activation measurement (24 h post-transfection), the Caspase-Glo 3/7 reagent (G8092/G8093 kit; Promega, Madison WI, USA) was left in room temperature to thaw slowly. The HeLa cell plates were left to equilibrate at room temperature. In the meantime, 50 μL of liquid was removed from all wells to achieve a total volume of 100 μL/well, for caspase activation measurement optimization; 1:1 ratio of Caspase-Glo 3/7 reagent volume to sample volume. Moreover, non-transparent back seals (6005199; Perkin-Elmer) were put under each plate. Henceforth, each cell plate is prepared separately, allowing for a 5-min processing gap to ensure adequate time for caspase activation measurement readout by the luminometer; the Caspase-Glo 3/7 assay is sensitive in regard to the peak of luminescent signal time. Following the preparation of the Caspase-Glo 3/7 reagent according to manufacturer's instructions, 100 μL were added slowly per well by dispensing on the wall of the well to avoid making bubbles. MicroAmp optical adhesive film was put on the plate, and its contents were mixed gently at 350–500 rpm for 30 s using a plate shaker. Each plate was left to incubate at room temperature for exactly 1 h in the dark. Finally, the plates were measured for caspase activation using as luminometer an EnSight multimode plate reader (HH34000000; Perkin-Elmer).

Binding Affinity of Gapmers

Lyophilized RNA corresponding to the sequence regions of interest was purchased from Integrated DNA Technologies (IDT, Coralville, IA, USA) and dissolved according to vendor's instructions to 100 μM final concentration. Equal amounts of gapmer and RNA corresponding to the target regions A or B, to which each gapmer binds, were dissolved in RNase/DNase free water (10977-035; Invitrogen) and dissolved in 2× T_m buffer (10 mM phosphate buffer, 100 mM NaCl, 0.1 mM EDTA [pH 7.0]) at a 1:1 ratio, to achieve a final concentration of 1.5 μM. Samples were denatured at 95°C for 3 min and subsequently left at room temperature to anneal by slowly cooling for 30 min and were finally spun down shortly. Thermal melting curves were logged at 260 nm on a Cary 100 UV-vis spectrophotometer using Cary WinUV software (G9821A; Agilent Technologies) equipped with a Cary temperature controller (G9844; Agilent Technologies), using a temperature gradient that increased by 1°C per min from 25°C to 95°C and then decreased back to 25°C. First derivatives and the local maxima of dissociation and annealing were used to evaluate the duplex T_m, defined as the temperature at which half of the gapmers are in duplex with target RNA.

Statistical Analysis

All data were analyzed using the statistical programming language R.[42] Significance of differences between gapmer knockdown activities or caspase activations was assessed by Welch's unequal variances t test. Significance of differences in T_m-optima was evaluated by nonparametric Wilcoxon rank-sum test. A linear model was used to relate the presence of 3-mer motifs found in the gap regions as well as ΔT_m, to the knockdown activity. Specifically, for each gapmer, each possible 3-mer motif was assigned either a 1 or a 0, depending on whether it was present or absent in the gap of that gapmer, and the T_m subtracted from the optimal T_m (see Table S1). To reduce sensitivity to outliers, model parameters were fitted by iteratively reweighted least-squares.[21] A random forests model[26] was used to relate dinucleotide motifs and ΔT_m to caspase activation levels. For each gapmer, the presence of each possible dinucleotide motif was counted and used as input to the model, and the T_m subtracted from the optimal T_m.

SUPPLEMENTAL INFORMATION

Supplemental Information can be found online at https://doi.org/10.1016/j.omtn.2019.12.011.

AUTHOR CONTRIBUTIONS

N.P. designed, conducted, and validated experiments, analyzed data, and wrote the paper. M.P. conducted the experiments for measurements of melting temperatures and reviewed the paper. M.R.A. supervised the work and reviewed and edited the paper. T.K. conceived the project idea and reviewed and edited the paper. P.H.H. conceived the project idea, supervised the work, analyzed data with statistical and machine-learning models, and wrote the paper.

CONFLICTS OF INTEREST

N.P., M.P., M.R.A., and T.K. declare they have no competing financial interests. P.H.H. is an employee of F. Hoffmann-La Roche, a company developing RNA-targeted therapeutics.

ACKNOWLEDGMENTS

For their excellent technical assistance, we would like to thank Charlotte Øverup and Annette Glidov. Additionally, we are grateful to Helle Christiansen, Bettina Nordbo, Dorthe Larsen, Henrik F. Hansen, Sanna L. Diemer, Christoph Rosenbohm, and Łukasz J. Kiełpiński for valuable discussions and support in the preparation of the manuscript. This research was supported with a stipend to N.P. from the Technical University of Denmark.

REFERENCES

1. Hughes, J.P., Rees, S., Kalindjian, S.B., and Philpott, K.L. (2011). Principles of early drug discovery. Br. J. Pharmacol. *162*, 1239–1249.
2. Sauer, W.H.B., and Schwarz, M.K. (2003). Molecular shape diversity of combinatorial libraries: a prerequisite for broad bioactivity. J. Chem. Inf. Comput. Sci. *43*, 987–1003.
3. Bleicher, K.H., Böhm, H.-J., Müller, K., and Alanine, A.I. (2003). Hit and lead generation: beyond high-throughput screening. Nat. Rev. Drug Discov. *2*, 369–378.
4. Hagedorn, P.H., Persson, R., Funder, E.D., Albæk, N., Diemer, S.L., Hansen, D.J., Møller, M.R., Papargyri, N., Christiansen, H., Hansen, B.R., et al. (2018). Locked nucleic acid: modality, diversity, and drug discovery. Drug Discov. Today *23*, 101–114.
5. Zamecnik, P.C., and Stephenson, M.L. (1978). Inhibition of Rous sarcoma virus replication and cell transformation by a specific oligodeoxynucleotide. Proc. Natl. Acad. Sci. USA *75*, 280–284.
6. Crooke, S.T., Witztum, J.L., Bennett, C.F., and Baker, B.F. (2018). RNA-Targeted Therapeutics. Cell Metab. *27*, 714–739.
7. Walder, R.Y., and Walder, J.A. (1988). Role of RNase H in hybrid-arrested translation by antisense oligonucleotides. Proc. Natl. Acad. Sci. USA *85*, 5011–5015.
8. Wu, H., Lima, W.F., Zhang, H., Fan, A., Sun, H., and Crooke, S.T. (2004). Determination of the role of the human RNase H1 in the pharmacology of DNA-like antisense drugs. J. Biol. Chem. *279*, 17181–17189.
9. Freier, S.M., and Watt, A.T. (2007). Basic principles of antisense drug discovery. In Antisense Drug Technology, S.T. Crooke, ed. (Springer), pp. 117–141.
10. Laxton, C., Brady, K., Moschos, S., Turnpenny, P., Rawal, J., Pryde, D.C., Sidders, B., Corbau, R., Pickford, C., and Murray, E.J. (2011). Selection, optimization, and pharmacokinetic properties of a novel, potent antiviral locked nucleic acid-based antisense oligomer targeting hepatitis C virus internal ribosome entry site. Antimicrob. Agents Chemother. *55*, 3105–3114.
11. Stanton, R., Sciabola, S., Salatto, C., Weng, Y., Moshinsky, D., Little, J., Walters, E., Kreeger, J., DiMattia, D., Chen, T., et al. (2012). Chemical modification study of antisense gapmers. Nucleic Acid Ther. *22*, 344–359.
12. Østergaard, M.E., Southwell, A.L., Kordasiewicz, H., Watt, A.T., Skotte, N.H., Doty, C.N., Vaid, K., Villanueva, E.B., Swayze, E.E., Bennett, C.F., et al. (2013). Rational design of antisense oligonucleotides targeting single nucleotide polymorphisms for potent and allele selective suppression of mutant Huntingtin in the CNS. Nucleic Acids Res. *41*, 9634–9650.
13. Greenberger, L.M., Horak, I.D., Filpula, D., Sapra, P., Westergaard, M., Frydenlund, H.F., Albaek, C., Schrøder, H., and Ørum, H. (2008). A RNA antagonist of hypoxia-inducible factor-1alpha, EZN-2968, inhibits tumor cell growth. Mol. Cancer Ther. *7*, 3598–3608.
14. Westergaard, M., Thrue, C.A., Rasmussen, F.W. and Hansen, H.F. (2009) WO/2006/050734; PCT/DK2005/000721. https://patentscope.wipo.int/search/en/detail.jsf?docId=WO2006050734
15. Thrue, C.A., Høg, A.M. and Kristjansen, P.E.G. (2010) WO/2006/050734; PCT/DK2005/000721. https://patentscope.wipo.int/search/en/detail.jsf?docId=WO2003085110.
16. McTigue, P.M., Peterson, R.J., and Kahn, J.D. (2004). Sequence-dependent thermodynamic parameters for locked nucleic acid (LNA)-DNA duplex formation. Biochemistry *43*, 5388–5405.
17. Pedersen, L., Hagedorn, P.H., Lindholm, M.W., and Lindow, M. (2014). A Kinetic Model Explains Why Shorter and Less Affine Enzyme-recruiting Oligonucleotides Can Be More Potent. Mol. Ther. Nucleic Acids *3*, e149.
18. Yamamoto, T., Fujii, N., Yasuhara, H., Wada, S., Wada, F., Shigesada, N., Harada-Shiba, M., and Obika, S. (2014). Evaluation of multiple-turnover capability of locked nucleic acid antisense oligonucleotides in cell-free RNase H-mediated antisense reaction and in mice. Nucleic Acid Ther. *24*, 283–290.
19. Cerritelli, S.M., and Crouch, R.J. (2009). Ribonuclease H: the enzymes in eukaryotes. FEBS J. *276*, 1494–1505.
20. Kiełpiński, Ł.J., Hagedorn, P.H., Lindow, M., and Vinther, J. (2017). RNase H sequence preferences influence antisense oligonucleotide efficiency. Nucleic Acids Res. *45*, 12932–12944.
21. Venables, W.N., and Ripley, B.D. (2013). Modern Applied Statistics with S-Plus (New York: Springer).
22. Dieckmann, A., Hagedorn, P.H., Burki, Y., Brügmann, C., Berrera, M., Ebeling, M., Singer, T., and Schuler, F. (2018). A Sensitive In Vitro Approach to Assess the Hybridization-Dependent Toxic Potential of High Affinity Gapmer Oligonucleotides. Mol. Ther. Nucleic Acids *10*, 45–54.
23. Fawcett, T. (2006). An introduction to ROC analysis. Pattern Recognit. Lett. *27*, 861–874.
24. Hagedorn, P.H., Hansen, B.R., Koch, T., and Lindow, M. (2017). Managing the sequence-specificity of antisense oligonucleotides in drug discovery. Nucleic Acids Res. *45*, 2262–2282.
25. Hagedorn, P.H., Yakimov, V., Ottosen, S., Kammler, S., Nielsen, N.F., Høg, A.M., Hedtjärn, M., Meldgaard, M., Møller, M.R., Ørum, H., et al. (2013). Hepatotoxic potential of therapeutic oligonucleotides can be predicted from their sequence and modification pattern. Nucleic Acid Ther. *23*, 302–310.
26. Breiman, L. (2001). Random Forests. Mach. Learn. *45*, 5–32.
27. Kurreck, J., Wyszko, E., Gillen, C., and Erdmann, V.A. (2002). Design of antisense oligonucleotides stabilized by locked nucleic acids. Nucleic Acids Res. *30*, 1911–1918.
28. Frieden, M., Christensen, S.M., Mikkelsen, N.D., Rosenbohm, C., Thrue, C.A., Westergaard, M., Hansen, H.F., Ørum, H., and Koch, T. (2003). Expanding the design horizon of antisense oligonucleotides with alpha-L-LNA. Nucleic Acids Res. *31*, 6365–6372.
29. Rouskin, S., Zubradt, M., Washietl, S., Kellis, M., and Weissman, J.S. (2014). Genome-wide probing of RNA structure reveals active unfolding of mRNA structures in vivo. Nature *505*, 701–705.
30. Vickers, T.A., Wyatt, J.R., and Freier, S.M. (2000). Effects of RNA secondary structure on cellular antisense activity. Nucleic Acids Res. *28*, 1340–1347.
31. Hentze, M.W., Castello, A., Schwarzl, T., and Preiss, T. (2018). A brave new world of RNA-binding proteins. Nat. Rev. Mol. Cell Biol. *19*, 327–341.
32. Shen, W., De Hoyos, C.L., Migawa, M.T., Vickers, T.A., Sun, H., Low, A., Bell, T.A., 3rd, Rahdar, M., Mukhopadhyay, S., Hart, C.E., et al. (2019). Chemical modification

of PS-ASO therapeutics reduces cellular protein-binding and improves the therapeutic index. Nat. Biotechnol. *37*, 640–650.

33. Muller, P.Y., and Milton, M.N. (2012). The determination and interpretation of the therapeutic index in drug development. Nat. Rev. Drug Discov. *11*, 751–761.
34. Juliano, R.L. (2016). The delivery of therapeutic oligonucleotides. Nucleic Acids Res. *44*, 6518–6548.
35. Khvorova, A., and Watts, J.K. (2017). The chemical evolution of oligonucleotide therapies of clinical utility. Nat. Biotechnol. *35*, 238–248.
36. Stein, C.A., Hansen, J.B., Lai, J., Wu, S., Voskresenskiy, A., Høg, A., Worm, J., Hedtjärn, M., Souleimanian, N., Miller, P., et al. (2010). Efficient gene silencing by delivery of locked nucleic acid antisense oligonucleotides, unassisted by transfection reagents. Nucleic Acids Res. *38*, e3.
37. Matveeva, O.V., Tsodikov, A.D., Giddings, M., Freier, S.M., Wyatt, J.R., Spiridonov, A.N., Shabalina, S.A., Gesteland, R.F., and Atkins, J.F. (2000). Identification of sequence motifs in oligonucleotides whose presence is correlated with antisense activity. Nucleic Acids Res. *28*, 2862–2865.
38. Chalk, A.M., and Sonnhammer, E.L.L. (2002). Computational antisense oligo prediction with a neural network model. Bioinformatics *18*, 1567–1575.
39. Camps-Valls, G., Chalk, A.M., Serrano-López, A.J., Martín-Guerrero, J.D., and Sonnhammer, E.L.L. (2004). Profiled support vector machines for antisense oligonucleotide efficacy prediction. BMC Bioinformatics *5*, 135.
40. Mergny, J.L., and Lacroix, L. (2003). Analysis of thermal melting curves. Oligonucleotides *13*, 515–537.
41. Chaires, J.B. (2008). Calorimetry and thermodynamics in drug design. Annu. Rev. Biophys. *37*, 135–151.
42. R Core Team (2018). R: A language and environment for statistical computing (Vienna, Austria: R Foundation for Statistical Computing), https://www.R-project.org/.

OMTN, Volume *19*

Supplemental Information

Chemical Diversity of Locked Nucleic Acid-Modified Antisense Oligonucleotides Allows Optimization of Pharmaceutical Properties

Natalia Papargyri, Malene Pontoppidan, Mikael R. Andersen, Troels Koch, and Peter H. Hagedorn

SUPPLEMENTARY FIGURES

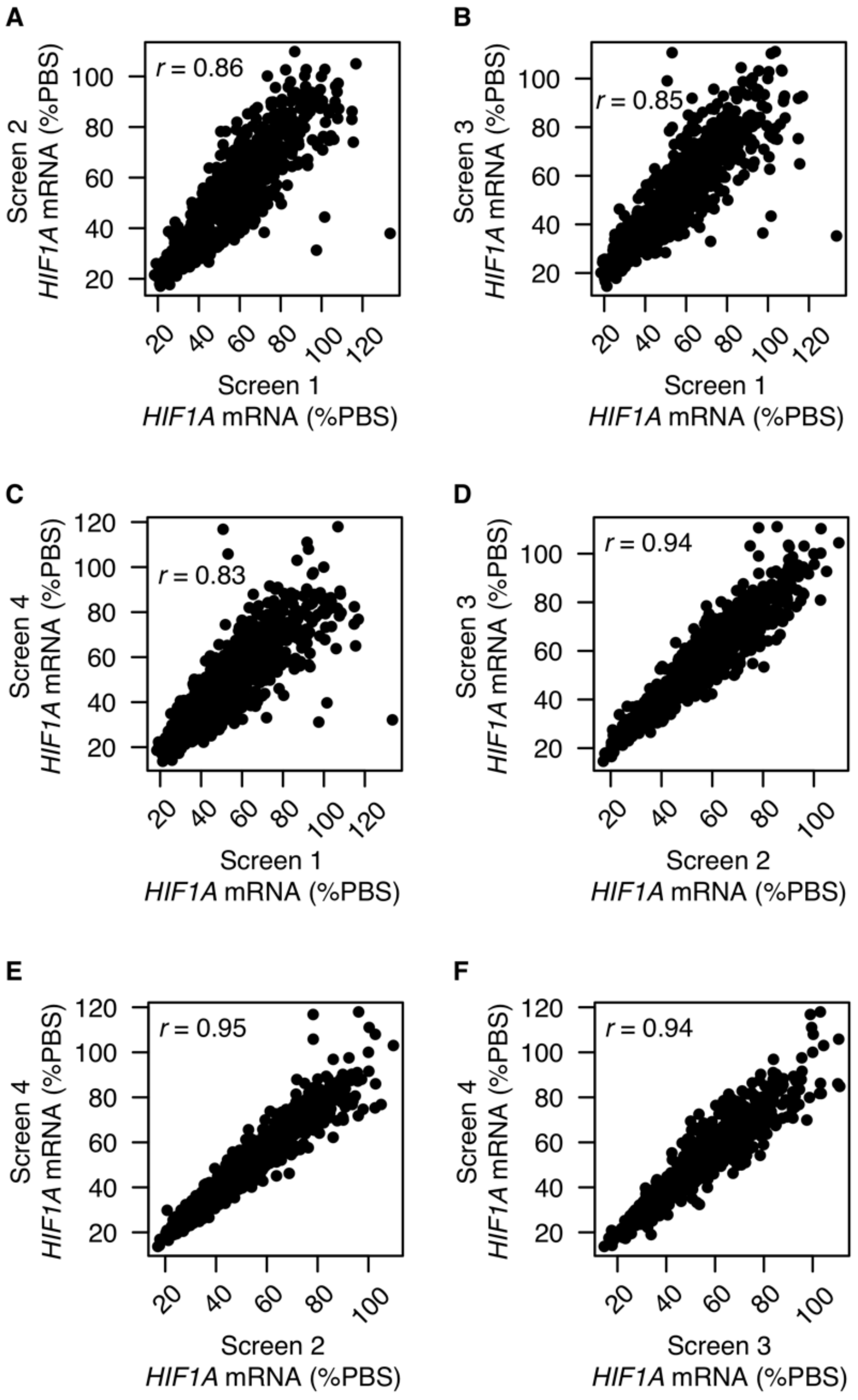

Figure S1. *Reproducible knockdown of HIF1A mRNA across four biological replicate screens*. All 768 LNA-gapmers evaluated in this study are shown. HeLa cells were treated for three days at a concentration of 5 µM by unassisted uptake and mRNA evaluated by RT-qPCR **A)** Screen 1 vs screen 2. **B)** Screen 1 vs screen 3. **C)** Screen 1 vs screen 4. **D)** Screen 2 vs screen 3. **E)** Screen 2 vs screen 4. **F)** Screen 3 vs screen 4. For all scatterplots, *r* indicates Pearson's correlation.

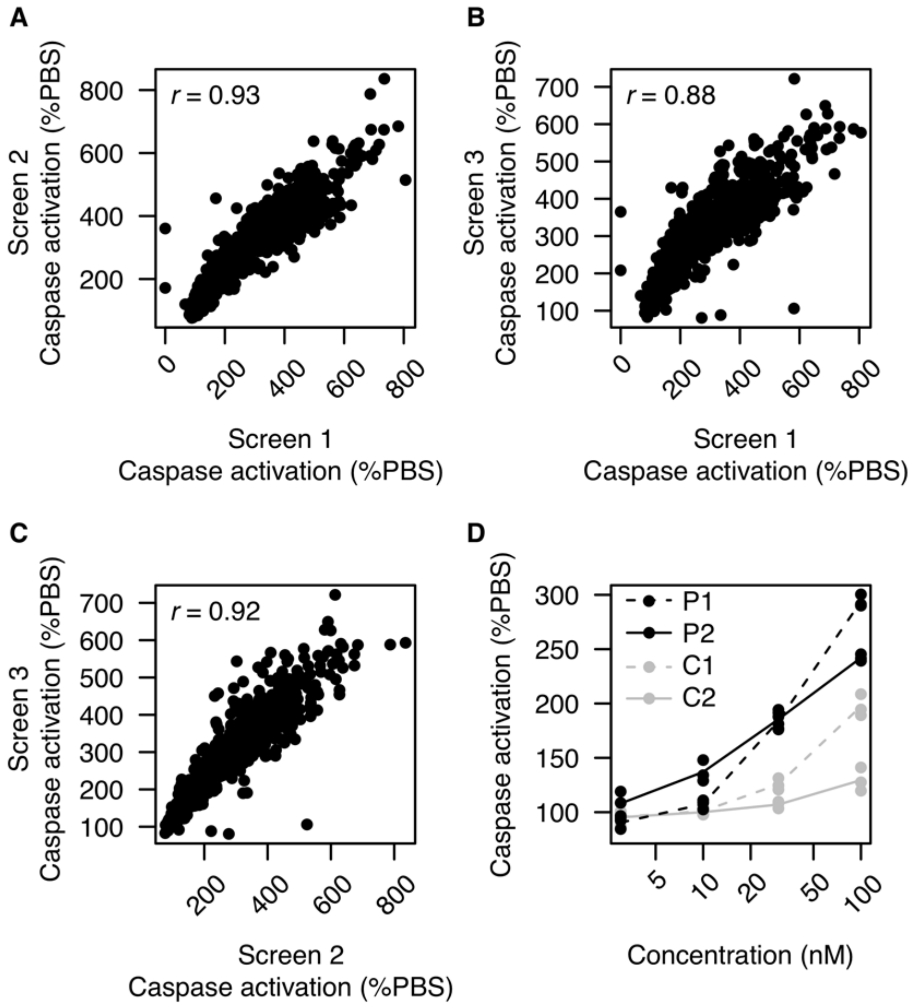

Figure S2. *Reproducible knockdown of caspase activation across three biological replicate screens and evaluation of concentration dependence.* **A)** Screen 1 vs screen 2. **B)** Screen 1 vs screen 3. **C)** Screen 2 vs screen 3. For all scatterplots ABC, *r* indicates Pearson's correlation, and all 768 LNA-gapmers evaluated in this study 24h after transfection of 100 nM in HeLa cells are shown. **D)** Evaluation of caspase activation of two positive control gapmers targeting *HIF1A* and known to knock down *HIF1A* mRNA, P1 and P2, and two negative control gapmers not targeting *HIF1A* and expected to be well-tolerated in cells C1 and C2, see Supplementary Table 1. Four concentrations tested 3, 10, 30 and 100nM. Points indicate individual measurements (*n*=3) and lines indicate average values.

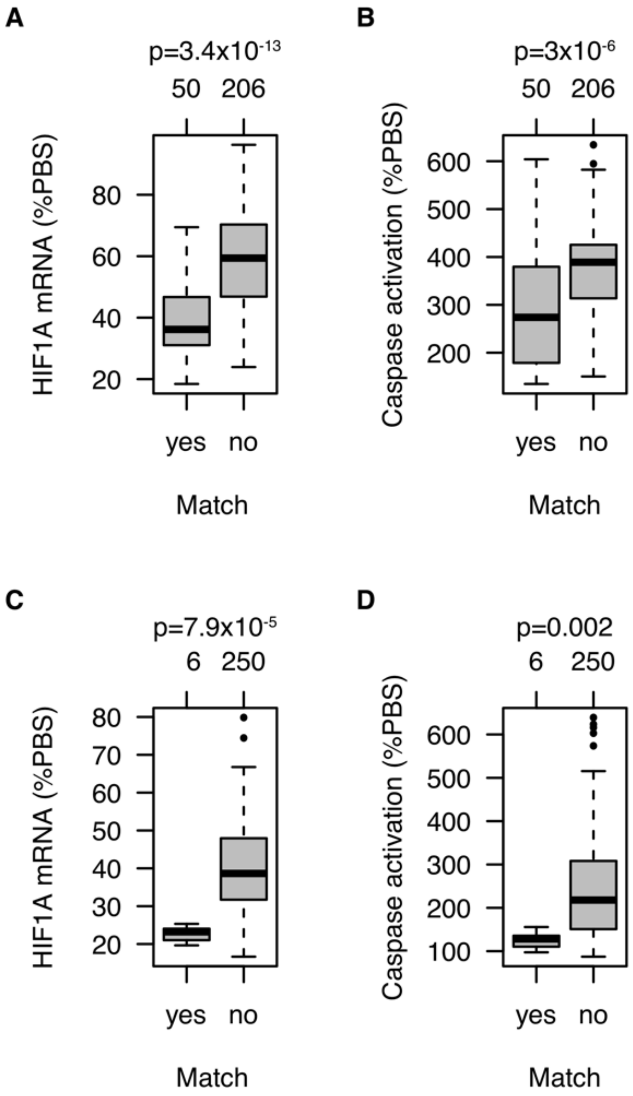

Figure S3. *Preferred chemical modification architectures.* **A)** For region A, boxplots of gapmers matching the preferred criteria (*n*=50 gapmers) with respect to knockdown activity and **B)** cytotoxic potential versus gapmers not matching those criteria (*n*=206). **C)** For region B, boxplots of gapmers matching the preferred criteria (*n*=6 gapmers) with respect to knockdown activity and **D)** cytotoxic potential versus gapmers not matching those criteria (*n*=250). Significance evaluated by two-sided Wilcoxon rank sum test.

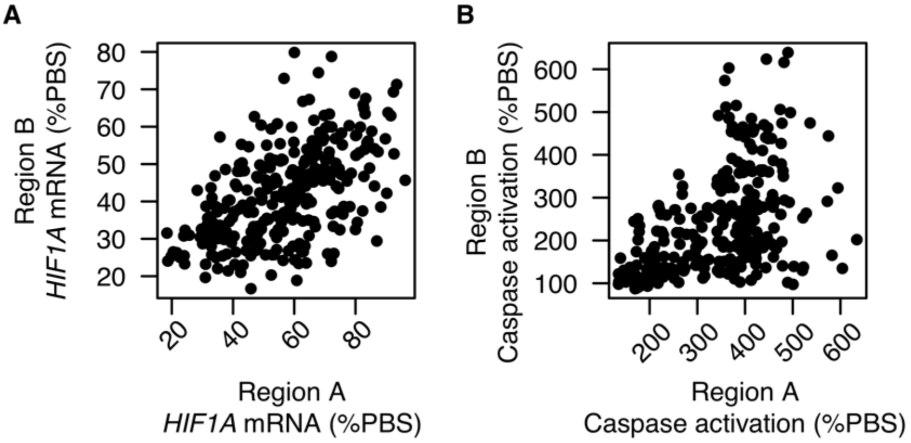

Figure S4. *Comparison of modification architectures between region A and B*. **A)** Knockdown activity. **B)** Cytotoxic potential.

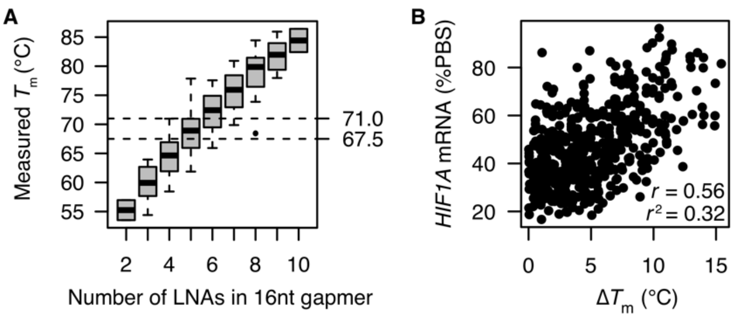

Figure S5. *Impact of LNAs on T_m, and T_m on knockdown activities.* **A)** Boxplots of T_m for 16nt gapmers as a function of the number of LNAs in each gapmer. Dashed lines indicate optimal T_m for regions A and B. **B)** Scatterplot of ΔT_m (absolute difference between gapmer T_m and optimal T_m in target region) versus knockdown activity. *r* indicates Pearson's correlation. r^2 indicates coefficient of determination.

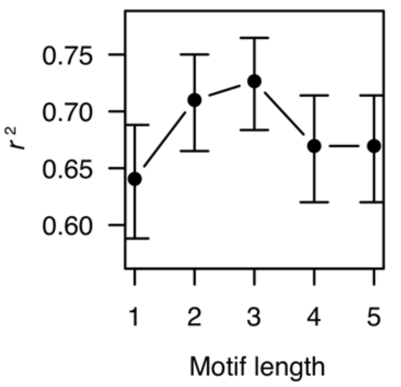

Figure S6. *Proportion of variance explained of knockdown activity of 16nt gapmers by robust linear model as a function of gap motif length in nt.* Error bars indicate 95% confidence intervals.

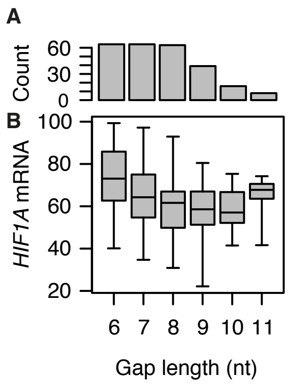

Figure S7. *Measured and predicted knockdown activities for 13nt gapmers stratified by gap sizes*. **A)** Barplot showing number of 13nt gapmers with gap sizes from 6 to 11nt. **B)** Boxplots of measured knockdown activities of *HIF1A* mRNA (%PBS) for 13nt gapmers stratified by gap size.
